# Atrial fibrillation as the first clinical presentation of an adenoid cystic bronchial carcinoma

**DOI:** 10.1007/s12471-014-0565-5

**Published:** 2014-05-20

**Authors:** Rainer Knur, Judit Özse

**Affiliations:** Department of Cardiology and Angiology, Allgemeines Krankenhaus Viersen, Hoserkirchweg 63, 41747 Viersen, Germany

**Fig. 1:**
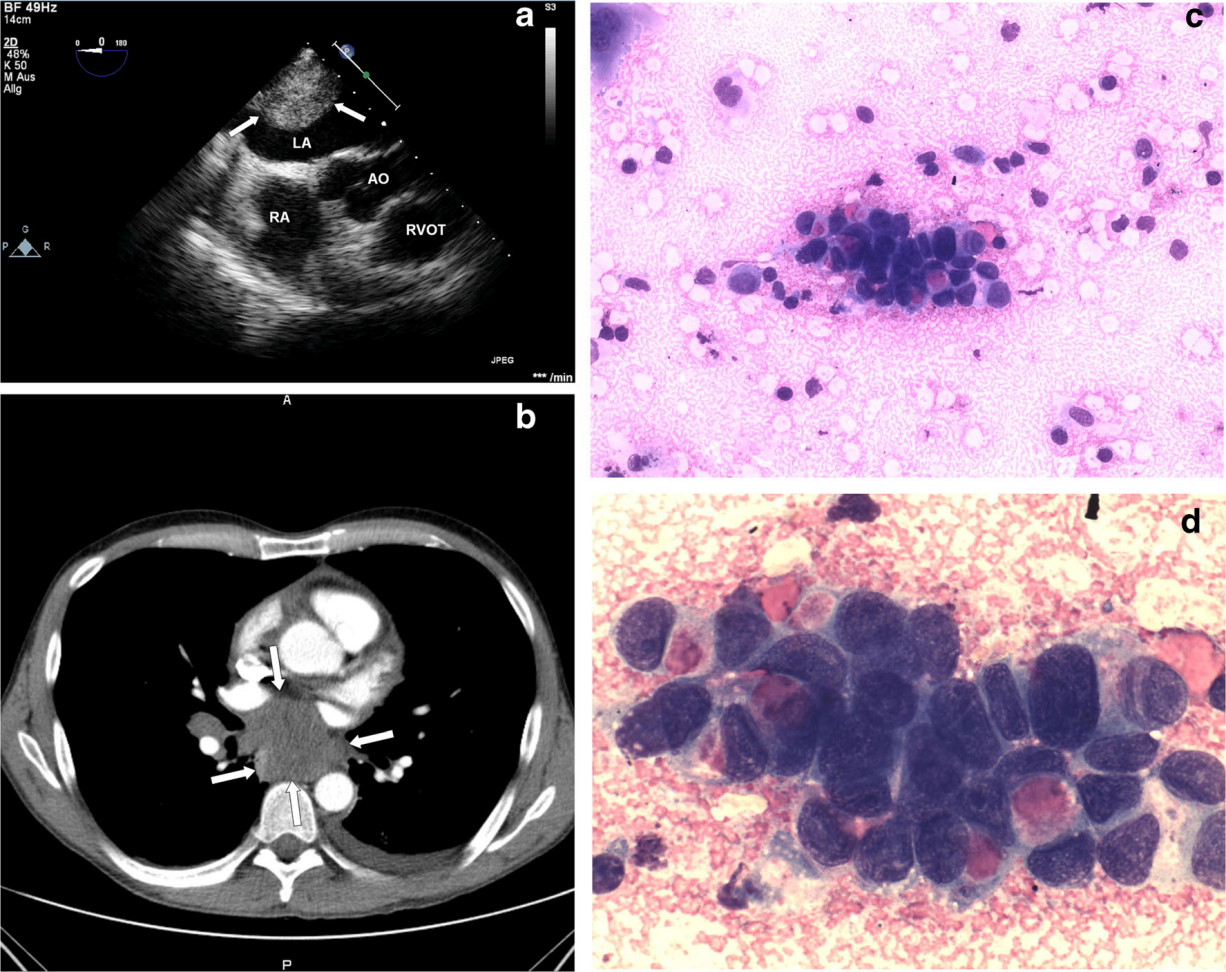
Transoesophageal echocardiogram (**a**) showing a left atrial mass (*arrows*). CT scan of the chest shows a polycyclic infracarinal tumour (*arrows*) with enlarged hilar lymph nodes and bilateral pleural effusion (**b**). An adenoid cystic bronchial carcinoma characteristically containing small, bland neoplastic cells and extracellular hyaline material was confirmed pathologically (**c**, **d**)

A previously healthy 50-year-old man was admitted with a repeated rapid heartbeat. Electrocardiograph on admission detected atrial fibrillation with an irregular ventricular rate of 170 beats/min for the first time. Transthoracic and transoesophageal echocardiography showed a 5 × 3.5 cm large sessile mass attached to the lateral wall of the left atrium (Fig. 1a) and a mild pericardial effusion. Computed tomography scan of the chest showed a lobulated lymphoma-like infracarinal mass 7.5 × 5 × 5 cm in size compromising the oesophagus and the left atrium, further enlarged hilar lymph nodes in the pathway of draining lymph ducts and bilateral pleural effusion (Fig. 1b). Cranial and abdominal CT was normal. An endobronchial ultrasound biopsy of the enlarged lymph nodes was performed. Histological and immunocytochemical examination confirmed an adenoid cystic bronchial carcinoma (Fig. 1c, d). The patient was immediately transferred to an oncology centre for further treatment.

Secondary cardiac neoplasms with an incidence of up to 1 % at autopsy are more than 20 times more common than primary cardiac tumours [1, 2]. The most common secondary malignant cardiac tumours including both metastasis and local extension were bronchial carcinoma, oesophageal carcinoma, carcinoma of the breast and lymphoma [3, 4]. Certain patients with cardiac neoplasms manifest only recurrent supraventricular or ventricular arrhythmias, most likely due to the irritative effect of tumour invading cardiac muscle [5]. Rarely, cardiac involvement may be the first clinical feature of malignancy. We present a case of an intrathoracic malignancy encroaching upon the heart and causing atrial fibrillation as its first presenting symptom.
